# Biocompatible exosome-modified fibrin gel accelerates the recovery of spinal cord injury by VGF-mediated oligodendrogenesis

**DOI:** 10.1186/s12951-022-01541-3

**Published:** 2022-08-02

**Authors:** Xiaolie He, Li Yang, Kun Dong, Feng Zhang, Yuchen Liu, Bei Ma, Youwei Chen, Jian Hai, Rongrong Zhu, Liming Cheng

**Affiliations:** Orthopaedics Department of Tongji Hospital, Key Laboratory of Spine and Spinal Cord Injury Repair and Regeneration of Ministry of Education, School of Medicine, School of Life Sciences and Technology, Tongji University, Shanghai, 200065 People’s Republic of China

**Keywords:** Exosomes, Fibrin gel, Spinal cord injury, VGF, Oligodendrogenesis

## Abstract

Exosomes show potential for treating patients with spinal cord injury (SCI) in clinical practice, but the underlying repair mechanisms remain poorly understood, and biological scaffolds available for clinical transplantation of exosomes have yet to be explored. In the present study, we demonstrated the novel function of Gel-Exo (exosomes encapsulated in fibrin gel) in promoting behavioural and electrophysiological performance in mice with SCI, and the upregulated neural marker expression in the lesion site suggested enhanced neurogenesis by Gel-Exo. According to the RNA-seq results, *Vgf* (nerve growth factor inducible) was the key regulator through which Gel-Exo accelerated recovery from SCI. VGF is related to myelination and oligodendrocyte development according to previous reports. Furthermore, we found that VGF was abundant in exosomes, and Gel-Exo-treated mice with high VGF expression indeed showed increased oligodendrogenesis. VGF was also shown to promote oligodendrogenesis both in vitro and in vivo, and lentivirus-mediated VGF overexpression in the lesion site showed reparative effects equal to those of Gel-Exo treatment in vivo. These results suggest that Gel-Exo can thus be used as a biocompatible material for SCI repair, in which VGF-mediated oligodendrogenesis is the vital mechanism for functional recovery.

**Supplementary Information:**

The online version contains supplementary material available at 10.1186/s12951-022-01541-3.

## Background

Spinal cord injury (SCI) is a kind of severe central nervous system (CNS) injury that results in impaired sensory and motor functions and a major burden on the patient's family and society [1]. In clinical practice, surgery or rehabilitation training has limited curative effects for patients with SCI and cannot effectively promote neural regeneration and functional recovery [2]. In recent years, the research development of exosomes and biological scaffolds has provided new hope for the treatment of SCI [3–5].

Exosomes with good biocompatibility are likely to play an important role in the clinical application of SCI [6, 7]. Exosomes can effectively promote functional recovery after SCI through their immunomodulatory, anti-inflammatory [8, 9], and antiapoptotic effects as well as their role in promoting vascular and axon regeneration [10, 11]. However, the specific mechanism is not clear and remains to be explored. Exosomes carry a variety of molecules, such as proteins, nucleic acids, and lipids [12, 13]. Approximately 4946 RNAs, 41,860 proteins, and 1116 lipids have already been found among different exosomes, according to the ExoCarta database [14]. Among these molecules, various neurotrophic factors in exosomes are very important [15, 16] but have rarely been studied. Therefore, unravelling the function of these neurotrophic factors will help us better understand the function of exosomes and thus allow them to be utilized in SCI.

Although researchers have focused on the insight that exosomes can promote the activation of axon regeneration, oligodendrogenesis is still an important contributor to the repair of SCI and the maintenance of spinal cord health [17]. Bobadilla et al. found that oligodendrogenesis from resident neural stem cells after SCI could promote axon remyelination and functional recovery of axon conduction [18]. Zhang et al. suggested that functional recovery post-SCI may be strongly correlated with Epo signaling-promoted oligodendrogenesis [19]. Similarly, Keirstead et al. demonstrated that engrafted oligodendrocyte precursor cells (OPCs) differentiated from human embryonic stem cells (ESCs) could facilitate the remyelination of the spinal cord and thus functionally improved motor deficits in rats with SCI [20]. In addition, recent studies have shown that treatment with miR-17-92 cluster-enriched exosomes had a notably stronger effect on enhancing oligodendrogenesis and functional recovery in stroke model rats [21]. Despite the finding that exosomes could promote oligodendrogenesis, the molecular pathways that drive SCI recovery still need further exploration.

Another issue that needs to be stressed is how to transplant exosomes into the injured site, and biological scaffolds provide available and diverse strategies. In animal models, alginate-based hydrogels could be employed as bioactive scaffolds to preserve exosomes at the wound site, which resulted in better wound closure, collagen synthesis, and angiogenesis [22]. Lei et al. demonstrated that exosome-loaded polyetheretherketone-based implant treatment not only promoted macrophage M2 polarization but also enhanced new bone formation as well as osseointegration in a rat femoral drilling model [23]. Many promising bioscaffold-based implantation techniques for exosome delivery have been developed in an attempt to restore function after neural injury but generally show unsatisfactory clinical results due to their poor biocompatibility. Hence, directly applying the biological scaffolds that have been used in clinical practice to exosome transplantation would be a potentially promising strategy.

**Scheme 1:**
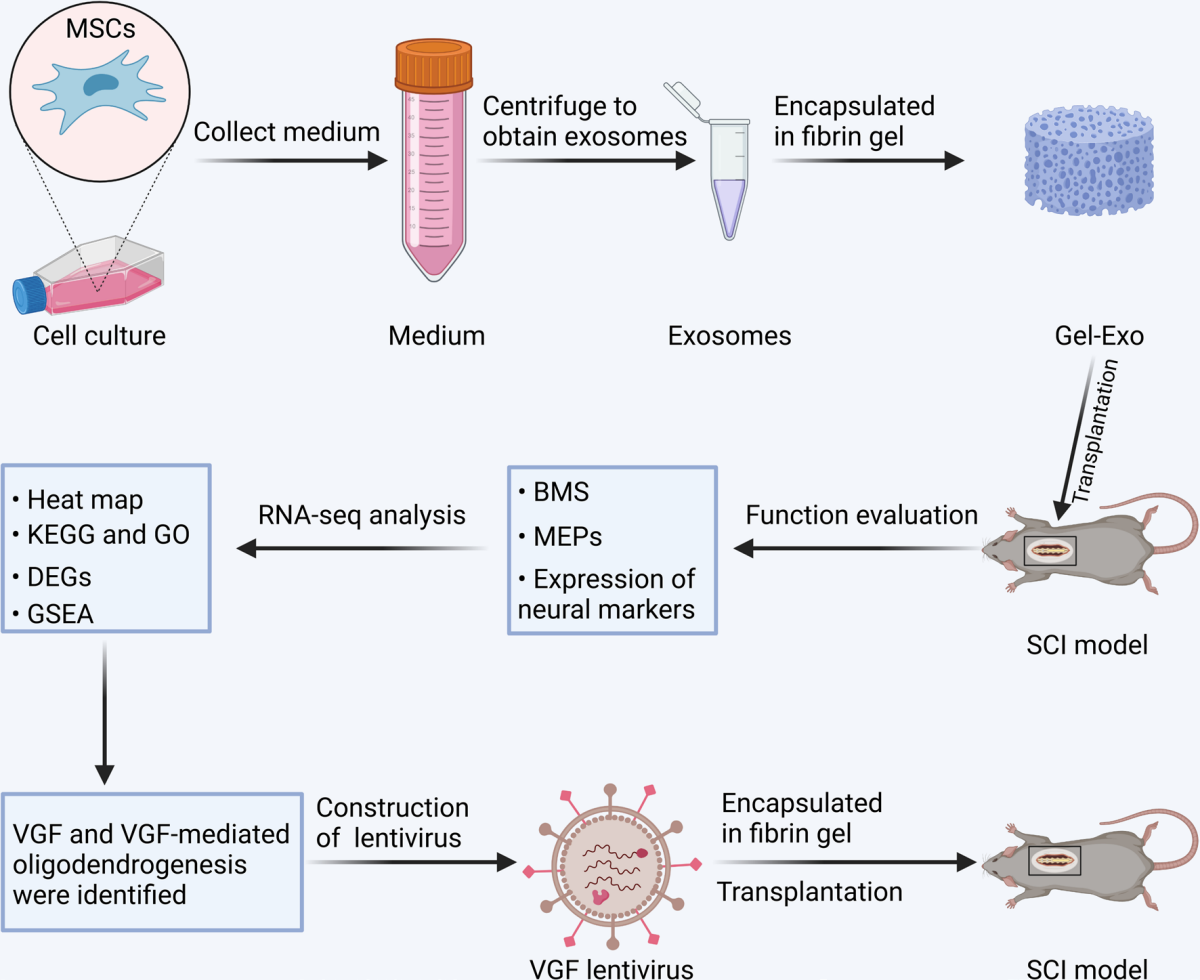
Sequential scheme of the experiments performed. Rat BMSC-derived exosomes were extracted and then encapsulated in fibrin gel to form Gel-Exo. The repair effects of Gel-Exo on SCI mice were investigated, and the underlying mechanisms were studied using RNA-seq analysis. VGF and VGF-mediated oligodendrogenesis were identified, then VGF lentivirus was employed to overexpress VGF in the lesion site of SCI, which accelerated the recovery of SCI. The figure was created with BioRender.com

In this study, we developed a novel biocompatible material for SCI repair using exosome-encapsulated fibrin gel (Gel-Exo), as fibrin gel is widely applied in clinical practice and has been approved by the FDA for its biocompatibility and wound healing properties [24, 25]. Here, we proved the novel function of Gel-Exo in promoting behavioural function recovery after injury, elucidated the molecular mechanism by transcriptome sequencing, and identified the neuropeptide precursor VGF (nerve growth factor inducible) as the main contributor to this effect. VGF was shown to be closely related to myelination and oligodendrocyte development [26], and OPCs express high levels of the *Vgf* gene [27]. Furthermore, we found that VGF was abundant in exosomes. Gel-Exo treatment with a high level of VGF indeed resulted in enhanced oligodendrogenesis. Overexpression of VGF promoted oligodendrogenesis in vitro and in vivo, and VGF lentivirus-mediated VGF overexpression in the lesion site showed an equal repair effect compared to Gel-Exo (Scheme 1). The major goal of this paper was to construct new biocompatible material comprising fibrin gel and exosomes or VGF lentivirus for SCI treatment. Furthermore, elucidating the underlying mechanism can provide a theoretical basis for the clinical application of exosomes.

## Results

### Identification and characterization of MSC-derived exosomes

**Fig. 1:**
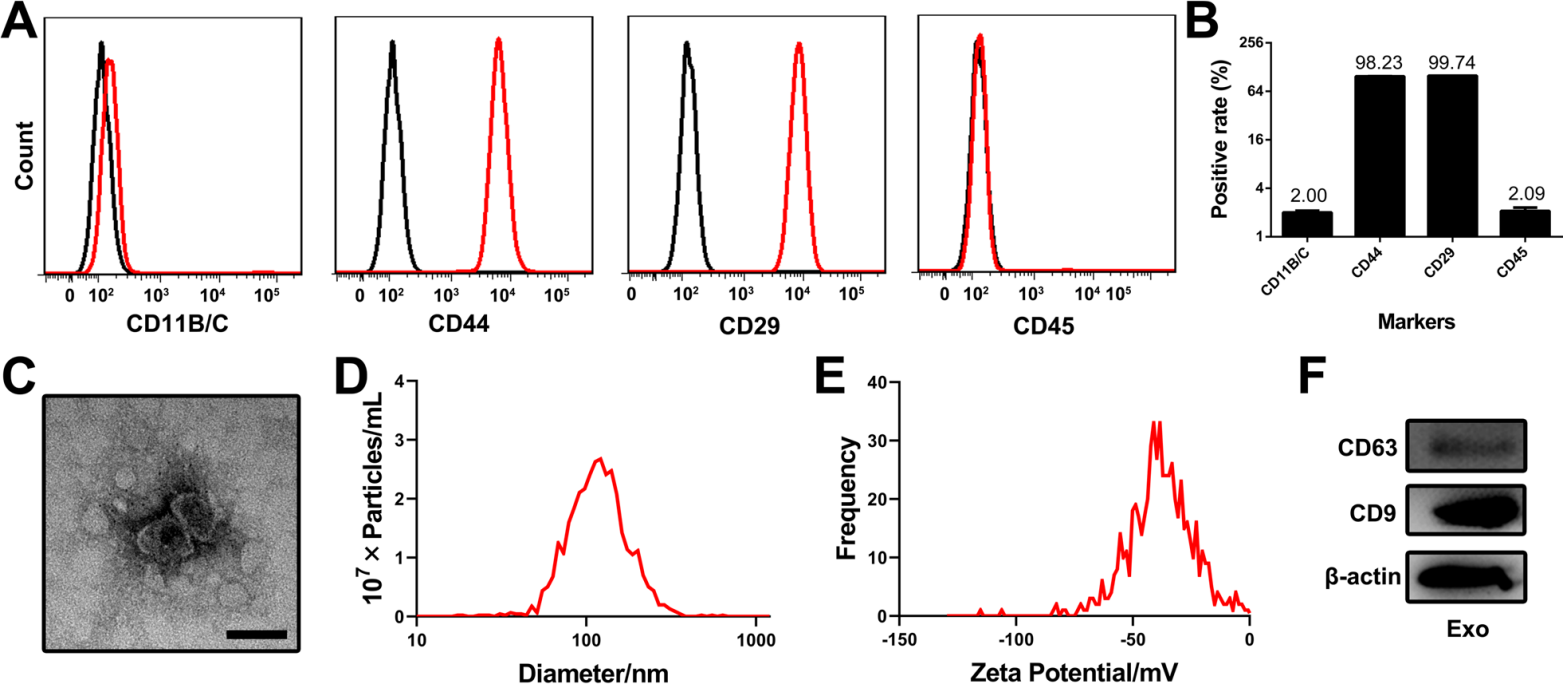
Characterization of BMSC-derived exosomes. **A** Identification of BMSCs by flow cytometry. **B** Quantification of positive rates for BMSC surface markers. **C** A representative TEM image of exosomes. Scale bar, 100 nm. **D** Size distribution assessment of exosomes by NTA. **E** Zeta potential analysis of exosomes by NTA. **F** Expression of CD63 and CD9 in exosome lysates analysed by Western blot

Rat bone marrow mesenchymal stem cells (BMSCs) were used to produce exosomes. To ensure cell purity, we detected the standard surface markers of MSCs by flow cytometry (Fig. 1A, B). The cells were strongly positive for CD44 and CD29 (positive rate > 98%) but negative for the haematopoietic markers CD11B/C and CD45 (positive rate < 3%), confirming that the cultured cells were highly pure MSCs. Exosomes were then extracted from the culture medium of MSCs by ultracentrifugation. Morphological examination under a transmission electron microscope (TEM) showed that the extracted exosomes displayed a classical saucer-like structure with a diameter approximately 100 nm (Fig. 1C). Moreover, the obtained exosomes were shown to have a mean diameter of 125.9 nm (Fig. 1D) and a negative zeta potential of − 37.66 mV (Fig. 1E) via nanoparticle tracking analysis (NTA). The expression of CD63 and CD9 (exosomal surface markers) was detected using Western blot analysis (Fig. 1F).

The above data demonstrated that MSC-derived exosomes were successfully extracted and exhibited typical exosome characteristics.

### Fibrin gel-embedded exosomes promoted functional recovery after SCI

**Fig. 2:**
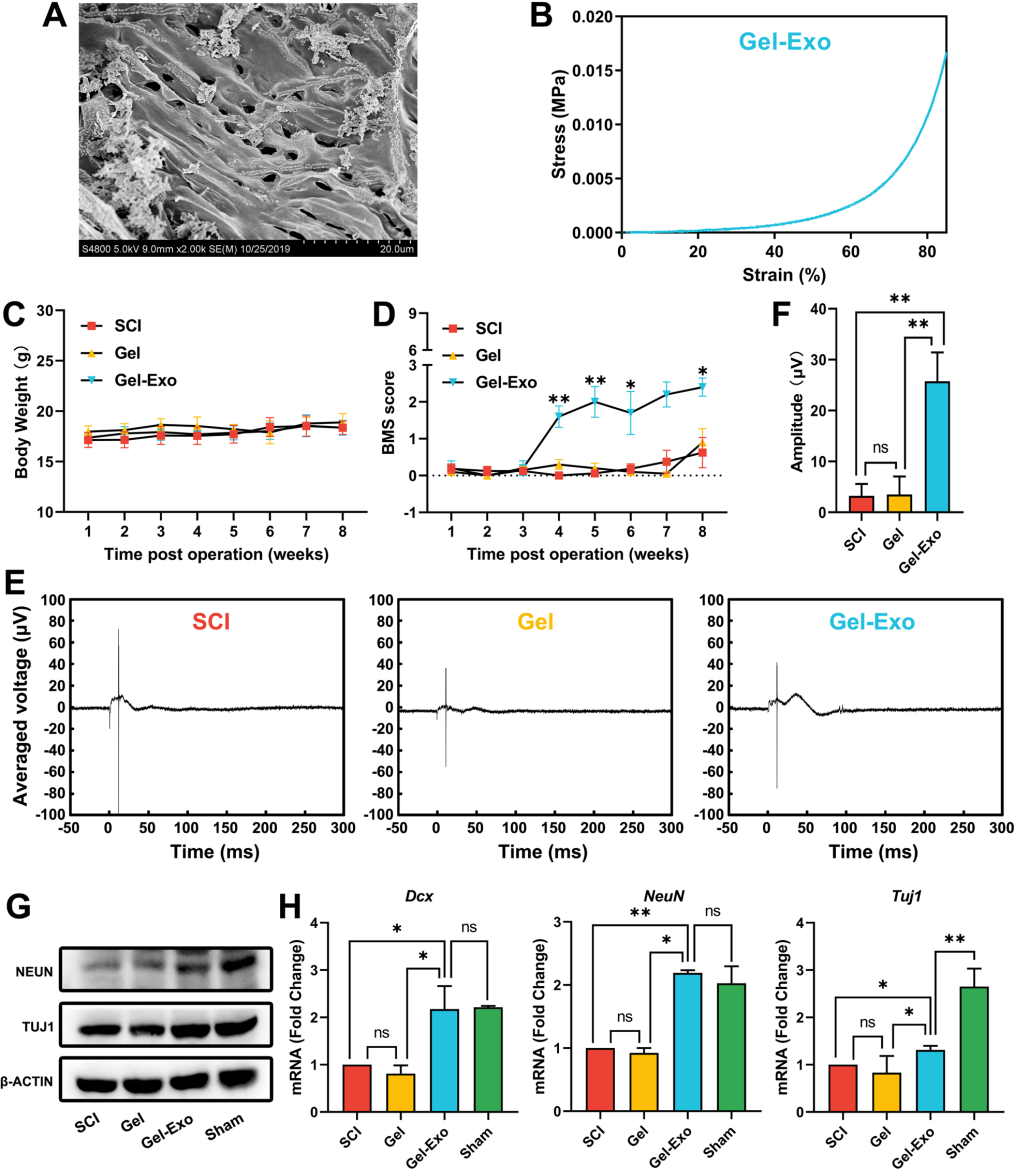
Gel-Exo facilitated recovery after complete SCI. **A** SEM analysis of Gel-Exo. **B** A representative stress–strain curve of Gel-Exo. **C** Body weight changes after the operation. **D** BMS open-field walking scores of mice with different treatments post-SCI over the course of 8 weeks. *p < 0.05 and **p < 0.01, when compared to the SCI group. **E** Electrophysiological analysis of the mice with different treatments after SCI. **F** The amplitude of MEPs; 3.23 ± 2.34, 3.52 ± 3.51, and 25.76 ± 5.65 μV for the SCI group, Gel group, and Gel-Exo group, respectively. *p < 0.05 and **p < 0.01, when compared to the SCI group or Gel group. **G**, **H** Expression of key neural markers in the injured spinal cord in each group. *p < 0.05 and **p < 0.01, when compared to the SCI group or sham group

Since aqueous exosomes have difficulty accumulating at the site of injury by traditional systemic administration methods, we employed fibrin gel, a natural hydrogel with good biocompatibility, to promote the immobilization of exosomes in the SCI lesion site. Fibrin gel (Gel) was prepared by mixing an equal volume of fibrinogen and thrombin solution, which mimicked the final step of the coagulation reaction. MSC-derived exosomes were incorporated into fibrin gels (Gel-Exo) for exosome delivery. Scanning electron microscopy (SEM) analysis of Gel-Exo demonstrated a suitable porous microstructure for cell infiltration and migration (Fig. 2A). Given that appropriate mechanical properties are vital for the survival and differentiation of neural cells [28], mechanical testing was performed, and the results showed that the compressive modulus of Gel-Exo was 1.212 kPa (Fig. 2B), which matched that of spinal tissue [29, 30].

We next explored whether Gel and Gel-Exo transplantation could potentiate functional recovery after complete spinal cord transection in mice. As shown in Fig. 2C, there was no significant change in body weight among the different treatment groups. Basso mouse scale (BMS) score analysis showed that the Gel-Exo-treated mice achieved significantly higher BMS scores from 4 weeks after SCI than the Gel-treated and nontreated mice (Fig. 2D). Notable motor functional recovery of the hindlimbs was observed in the Gel-Exo group 8 weeks post-injury, as indicated by a BMS score of 2.4 (Fig. 2D and Additional file 2: Video S1). However, no distinct difference was observed between the Gel group and the SCI group, which implied that Gel treatment alone would not have an obvious impact on injured mice. In addition, as depicted in Fig. 2E and F, Gel-Exo treatment greatly enhanced the motor evoked potential (MEP) amplitude at 8 weeks after SCI (25.76 μV), whereas no significant increase in MEP amplitude was detected in the Gel group.

To verify the effects of Gel-Exo on neurogenesis, we determined the expression levels of the neural markers neuronal nuclei (NEUN) and neuronal class III β-Tubulin (TUJ1) via western blots, which revealed that the levels of NEUN and TUJ1 were greatly elevated in the mice transplanted with Gel-Exo (Fig. 2G). Consistent with the protein expression, the mRNA levels of doublecortin (*Dcx*), *NeuN*, and *Tuj1* were markedly increased in the Gel-Exo group compared to the SCI and Gel groups (Fig. 2H). Notably, the expression levels of *Dcx* and *NeuN* in the Gel-Exo group were similar to those in the sham group, indicating the potential of Gel-Exo in promoting neural repair.

In summary, the as-prepared Gel-Exo composite with porous structure and appropriate stiffness could promote motor function and electrophysiological performance in mice with SCI, and the upregulated neural marker expression in the lesion site suggested enhanced neurogenesis by Gel-Exo. Our results demonstrated that fibrin-based exosome delivery could be a promising strategy for SCI therapy.

### RNA sequencing revealed the upregulation of neuropeptide VGF in Gel-Exo-transplanted mice

**Fig. 3:**
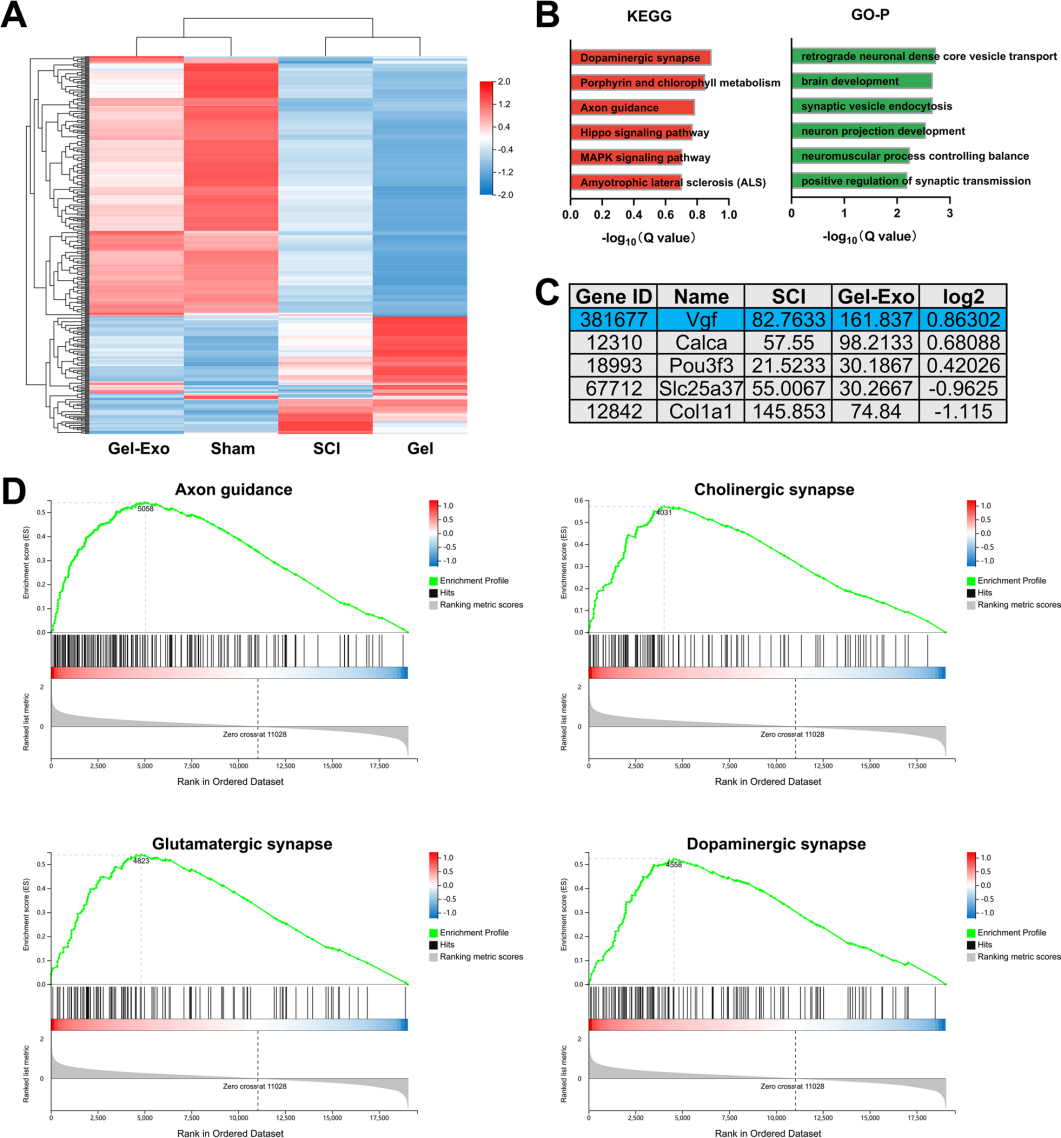
RNA-seq analysis of Gel-Exo-treated mice with SCI. **A** Hierarchical clustering heatmap of DEGs. **B** KEGG pathway and GO enrichment analyses. **C** The five most relevant DEGs between the spinal cords of the Gel-Exo group and SCI group. **D** GSEA KEGG pathway analysis between the Gel-Exo group and SCI group

Although exosomes are known to transfer various bioactive molecules, such as microRNAs and proteins, to recipient cells, the associated mechanism of exosome-mediated SCI repair still needs further exploration. Thus, RNA sequencing was performed to identify key pathways and genes involved by analysing the differences in transcriptome profiles between groups. The hierarchical clustering heatmap presented in Fig. 3A shows an obvious distinction in gene expression between the sham and SCI groups. Notably, the gene expression profile of the Gel-Exo group closely matched that of the sham group, while the Gel group was more similar to the SCI group. KEGG and GO enrichment analyses were conducted on differentially expressed genes (DEGs) between the Gel-Exo group and the SCI group (Fig. 3B). The DEGs were mainly enriched in dopaminergic synapse, axon guidance, Hippo signaling pathway, and mitogen-activated protein kinase (MAPK) signaling pathway and were involved in many neural-related biological processes, such as synaptic vesicle endocytosis and positive regulation of synaptic transmission.

Five genes (*Vgf*, *Calca*, *Pou3f3*, *Slc25a37*, and *Col1a1*) differentially expressed in the Gel-Exo group were manually screened according to the fold change values and literature reports (Fig. 3C). *Vgf* is closely related to myelination and oligodendrocyte development [26], which may be beneficial to the repair of SCI. *Calca* is a potent vasodilator [31] and plays a critical role in transmitting pain messages to the spinal cord [32]. Activation of CALCA-expressing glutamatergic neurons in the midbrain perioculomotor region could promote non-rapid eye movement (NREM) sleep [33]. In addition, CALCA-expressing interneurons in the spinal cord were found to be suppressed in the normal state and became hyperexcitable after nerve injury, which might contribute to mechanical allodynia [32]*. Pou3f3* is a neurogenic transcription factor that regulates various steps of neurogenesis [34]. It is also a cortical layer-specific marker (Layer II/III) and a key modulator of cell-intrinsic fate transition in the developing mouse cortex [35]. *Slc25a37* encodes a mitochondrial Fe ion transporter that exerts an essential function in iron homeostasis. This molecule has been associated with energy metabolism and spatial memory of the brain [36]. *Col1a1* encodes a protein that is the major component of type I collagen (Col I), which has been reported to be produced by pericytes and fibroblasts after SCI [37]. In particular, Col I is significantly expressed in the lesion area during the scar-forming phase following SCI and is involved in astrocytic scar formation, which is one of the barriers to SCI repair [37]. Among these genes, the neuropeptide precursor *Vgf* attracted our particular attention because it has been reported in many neural disorders, including Parkinson’s disease (PD) [38], Alzheimer’s disease (AD) [39], and major depressive disorder (MDD) [40]. However, the role of VGF in SCI has not yet been studied. *Vgf*, as a significant DEG, is thought to be involved in the Gel-Exo-mediated repair process, which will be further analysed in our study.

In addition, gene set enrichment analysis (GSEA) revealed a positive normalized enrichment score (NES) for gene sets associated with neural development and neurotransmission, such as Axon guidance (NES = 2.38), Cholinergic synapse (NES = 2.37), Glutamatergic synapse (NES = 2.27), and Dopaminergic synapse (NES = 2.23), suggesting that Gel-Exo treatment might enhance synaptic functions in mice with SCI (Fig. 3D).

### Gel-Exo increased VGF abundance in the lesion site

**Fig. 4:**
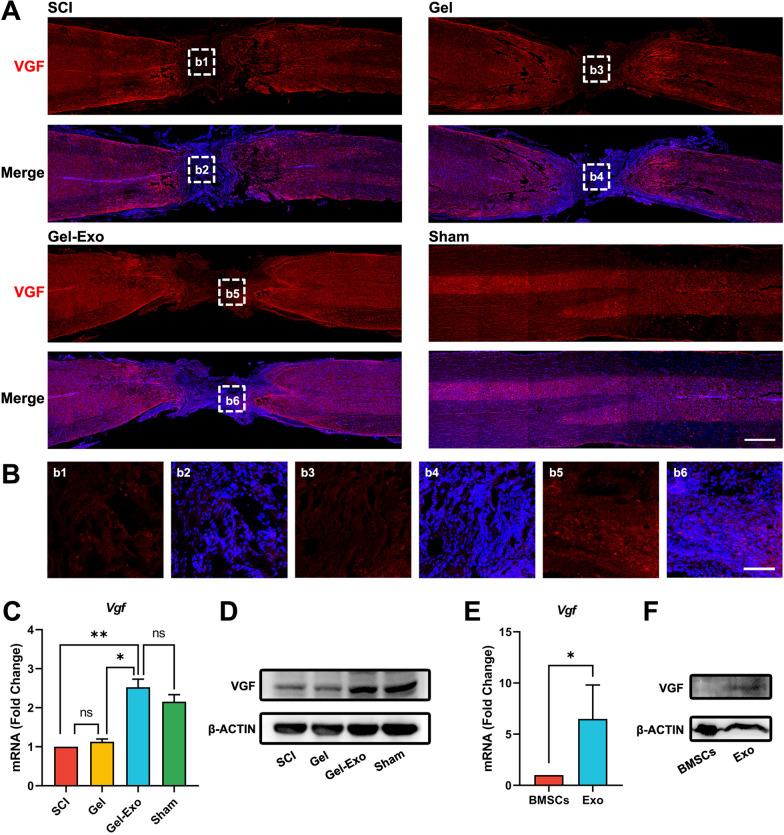
Gel-Exo increased VGF abundance in the lesion site. **A** Immunostaining of VGF in the mouse spinal cord. Scale bar, 1000 μm. **B** Amplified images of highlighted regions at the lesion site of mice with SCI. Scale bar, 100 μm. **C** qPCR validation of *Vgf* expression in various treatments of injured spinal cord. *p < 0.05 and **p < 0.01, when compared to the SCI group or sham group. **D** Western blot detection of VGF expression in the injured spinal cord. **E**, **F** Expression of *Vgf* mRNA and VGF protein in exosomes

We first validated VGF expression at the lesion site by immunostaining. A robust increase in fluorescence intensity was detected in the Gel-Exo-treated mice compared with the Gel-treated mice and the mice with SCI (Fig. 4A, B). The mRNA and protein expression of VGF was also significantly upregulated following Gel-Exo treatment (Fig. 4C, D), which was similar to the sham group. Furthermore, we found that VGF was abundant in secreted exosomes but had lower expression in the cellular proteome of BMSCs (Fig. 4E, F), indicating that the highly expressed VGF in the lesion site might be attributed to exosomal VGF.

In conclusion, our results demonstrated the significant upregulation of VGF in the lesion site after Gel-Exo treatment, and the increased VGF originated from fibrin gel-delivered exosomes.

### Gel-Exo promoted oligodendrogenesis in the lesion site after SCI

**Fig. 5:**
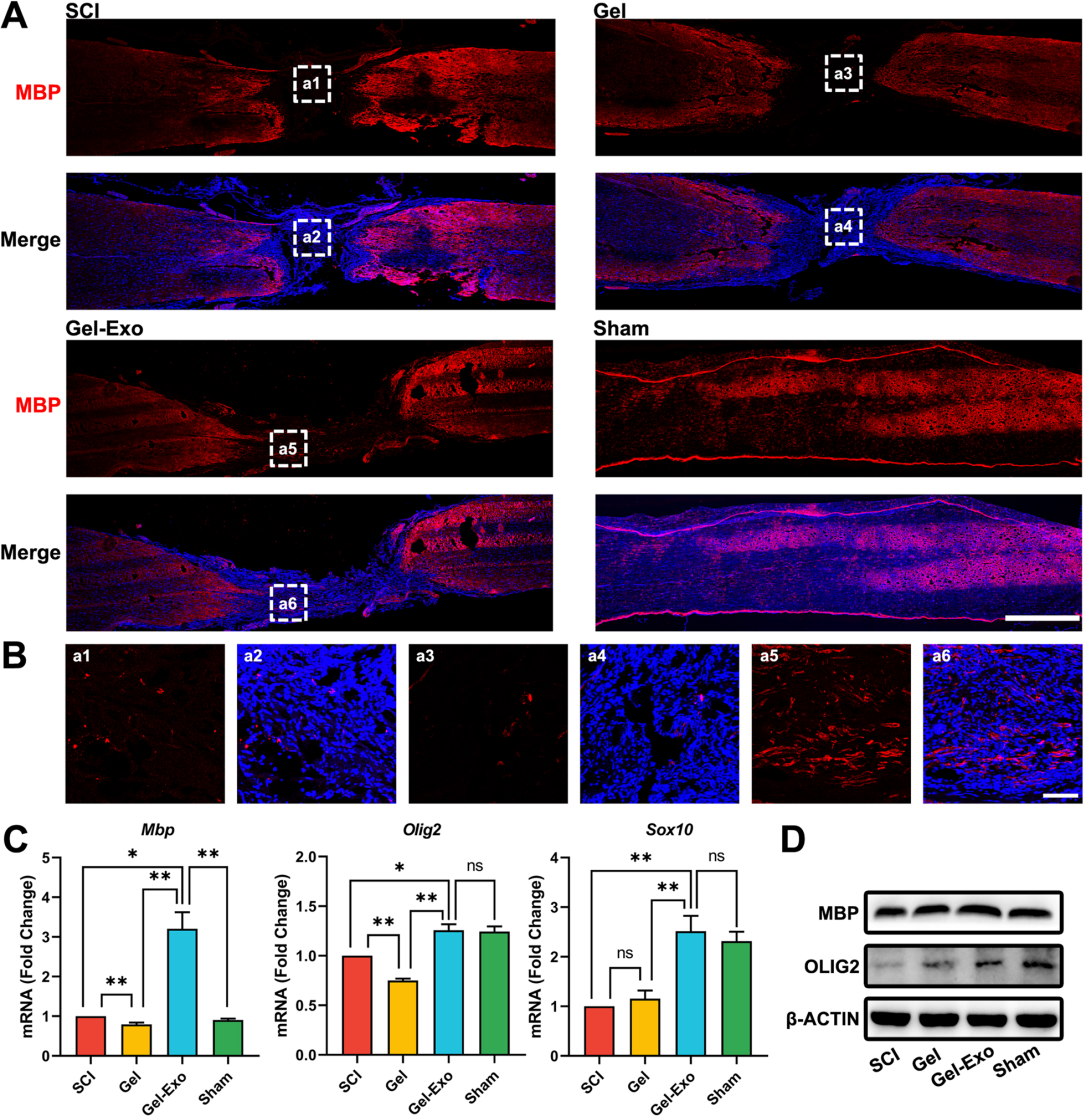
Gel-Exo promoted oligodendrogenesis in the lesion site after SCI. **A** Immunostaining of MBP in different groups. Scale bar, 1000 μm. **B** Amplified images of highlighted regions at the lesion site of SCI mice. Scale bar, 100 μm. **C**, **D** mRNA and protein expression of mature oligodendrocyte markers

VGF is closely linked with myelination and oligodendrocyte development [26, 27], and oligodendrogenesis is important for repairing SCI [18, 41]. Based on the results in Fig. 4, we investigated whether Gel-Exo-induced persistent expression of VGF in the lesion site would promote oligodendrogenesis. The expression of the mature oligodendrocyte (OL) marker myelin basic protein (MBP) was investigated in the spinal cord, especially in the lesion site, and only few MBP-positive cells were observed in the lesion site of the SCI or Gel group. However, many oligodendrocytes (OLs) were observed in the Gel-Exo group, which was due to the effects of VGF to a large extent (Fig. 5A, B). In addition, the mRNA expression of mature OL markers (*Mbp*, *Olig2*, and *Sox10*) in the lesioned spinal cord was detected via real-time quantitative PCR (qPCR). Gel-Exo resulted in a robust increase in *Mbp*, *Olig2*, and *Sox10* expression levels compared to SCI and Gel, almost identical to the sham group (Fig. 5C). Moreover, we performed Western blotting to confirm the protein levels of MBP and OLIG2, and the blot images of spinal cord samples from each group clearly showed that the expression of MBP and OLIG2 in the Gel-Exo and sham groups was markedly higher than that in the SCI group (Fig. 5D), which was consistent with what was proven by immunostaining and qPCR.

In summary, Gel-Exo treatment with persistent expression of VGF could induce oligodendrogenesis, which was beneficial for recovery from SCI.

### Overexpression of VGF promoted oligodendrogenesis in vitro and in vivo

The upregulation of VGF in the lesion site (Fig. 4A) drew our special attention because it has been reported that OPCs express high levels of VGF [27]. Furthermore, reduced VGF levels are connected with the demyelination process [42, 43]. To determine whether VGF overexpression was sufficient to promote oligodendrogenesis in vitro and restore the function of mice with SCI in vivo, we constructed lentiviral vectors expressing mouse VGF protein that could be utilized both in vitro and in vivo.

**Fig. 6:**
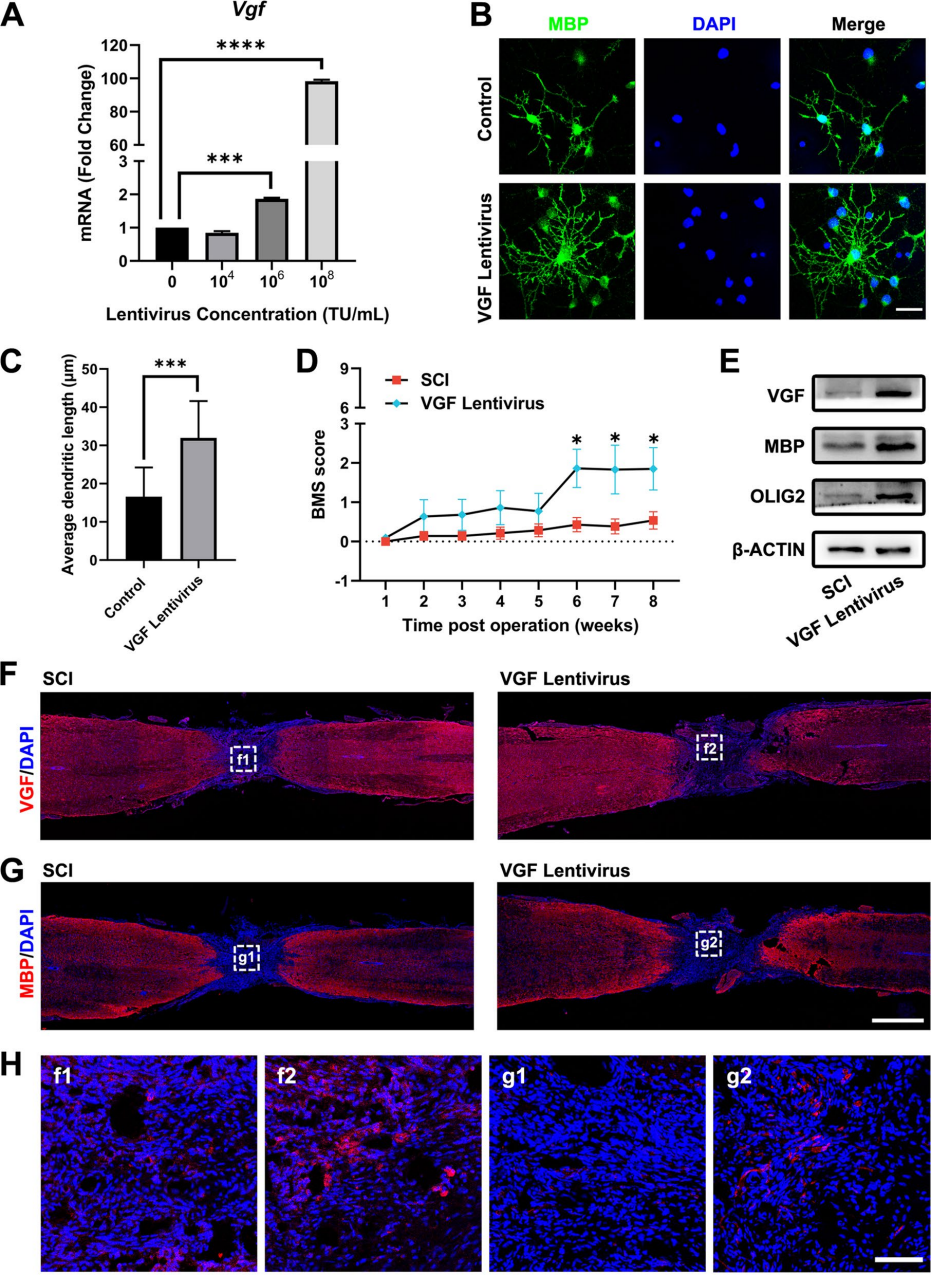
Overexpression of VGF promoted oligodendrogenesis in vitro and in vivo. **A** qPCR detection of *Vgf* after VGF overexpression in cells using various doses of lentivirus. **B** Immunostaining of MBP (green) under treatment with 10^8^ TU/mL VGF lentivirus. Nuclei were stained with DAPI (blue). Scale bar, 10 μm. **C** Quantification of the average dendritic length. **D** BMS open-field walking scores of the mice treated with VGF lentivirus for 8 weeks. *p < 0.05 compared to the SCI group. **E** Western blot detection of VGF, MBP, and OLIG2 expression in the injured spinal cord after VGF lentivirus treatment. **F**, **G** Staining of VGF and MBP in the lesioned spinal cord. Scale bar, 1000 μm. **H** Amplified images of highlighted regions at the lesion site of mice. Scale bar, 100 μm

In the in vitro experiments, we treated primary OPCs with various concentrations of VGF lentivirus to overexpress VGF and investigated the efficacy after the differentiation of OPCs. The results suggested that 10^8^ TU/mL particles could induce 98-fold higher VGF expression (Fig. 6A). Then, we stained differentiated cells with MBP and found that VGF lentivirus significantly increased the mean dendritic length of oligodendrocytes, indicating that VGF could stimulate the differentiation of OPCs into OLs (Fig. 6B, C).

In the in vivo experiments, mice in the VGF lentivirus group were transplanted with fibrin gel-embedded lentivirus at the lesion site, and the function of VGF was assessed. We recorded the BMS score after VGF treatment, and a significantly elevated score was detected in the VGF lentivirus group 8 weeks post-operation, which implied that VGF could restore the function of the mice with SCI (Fig. 6D and Additional file 3: Video S2). Using Western blots to analyse protein levels in the lesioned spinal cord of mice in the SCI and VGF groups, we found significant upregulation of the VGF, MBP, and OLIG2 proteins in the lesion site (Fig. 6E). Moreover, we observed increased expression of VGF and MBP in the lesion site through immunostaining (Fig. 6F, G, H).

Thus, overexpression of VGF could promote oligodendrogenesis both in vitro and in vivo, and lentivirus-mediated VGF overexpression in the lesion site showed equivalent repair effects compared to Gel-Exo treatment in vivo. Our data suggest that VGF is a key target for SCI therapy, which may also have potential in clinical transformation.

### Biosafety assessment of Gel-Exo treatment

Histological examination was carried out for each group mentioned above (including the SCI, Gel, Gel-Exo, VGF lentivirus, and sham groups) in our study. As shown in Additional file 1: Fig. S1, the morphology of all harvested organs in the Gel, Gel-Exo, and VGF lentivirus groups was as normal as that in the sham group, indicating that both Gel-Exo and VGF lentivirus treatments were safe enough, which provided preliminary data to support future clinical transformation.

## Discussion

Exosomes are rich in a large number of bioactive molecules, which exert many important functions both in vitro and in vivo. Meng et al. proved the presence of large amounts of granulocyte–macrophage colony stimulating factor (GM-CSF) in exosomes, which have the potential to enhance the immunomodulatory function [44]. Sun et al. demonstrated that patients with metastatic colorectal cancer had higher serum levels of exosome-derived ADAM17 and that exosomal ADAM17 could facilitate colorectal cancer cell migration by cleaving the E-cadherin junction [45]. Chen et al. showed that neuronal apoptosis induced by necroptotic astrocyte-derived exosomes could be dramatically impaired by blocking pro-BDNF (brain-derived neurotrophic factor), suggesting that necroptotic astrocytes provoked neuronal apoptosis partially through exosome-derived pro-BDNF [46]. Exosomes have shown potential in repairing SCI, and the function of exosomes is closely related to the bioactive molecules they contain, so it is vital to identify the key molecules that enable exosomes to perform their part in repairing SCI.

VGF has widespread influences on neurological diseases [40, 47–50]. Overexpression of VGF could improve memory performance and reduce neuropathology in mice with AD, supporting a causal role for VGF in AD pathogenesis and progression [39]. Winter et al. identified VGF as the top biomarker distinguishing familial PD [38]. Additionally, VGF was reported to be closely linked to myelination and oligodendrocyte development [26, 27]. According to our research, Gel-Exo treatment with high VGF expression resulted in more oligodendrogenesis (Fig. 5). We also demonstrated that lentivirus-mediated overexpression of VGF not only significantly promoted OPC differentiation into OLs using primary rat OPCs cultured in vitro but also improved functional recovery from SCI and triggered de novo myelination in the lesion site (Fig. 6).

Recently, biological scaffolds have opened a new door for the application of exosomes in neural regeneration and repair [51]. These biological scaffolds should meet the following requirements: (1) they are nontoxic and have potential in clinical applications; (2) exosomes can be loaded and released slowly within scaffolds; and (3) they have pores where cells can grow. Scaffolds, including alginate scaffolds, chitosan hydrogels, and chitin conduits, have been employed for nerve injury repair [52–54]. The materials used for SCI repairing can be mainly divided into natural biomaterials and synthetic biomaterials. Natural biomaterials mainly include decellularized spinal cord scaffolds, collagen, hyaluronic acid, and chitosan. These biomaterials are characterized with low mechanical strength and fast degradation rate, and their three-dimensional porous structure will be destructed after water absorption and swelling, which would not be beneficial for neuron regeneration. Synthetic biomaterials mainly include PCL, PLA, PLGA, and PEG. The disadvantages of these materials are that the degradation products would induce local inflammatory reactions and damage the local microenvironment, and eventually unfriendly to cell. To make cells adhere to the materials and better promote neural regeneration, it is a need to prepare a composite scaffold by combining natural materials and synthetic materials. Overall, future research is required to evaluate these materials from multi-aspects for clinical use. At this time, directly applying the biological scaffolds used in clinical practice for exosome transplantation would be a good potential choice.

Compared to the materials mentioned above, fibrin gel is an appropriate and ideal bio-scaffold with degradability and biocompatibility and can be applied in SCI repair [55–57]. Fibrin gel is a biodegradable polymer that can be degraded by the serine protease plasmin produced by cells. The process of degradation is controllable and can be modulated by the addition of plasmin inhibitors, such as aprotinin [55] or ɛ-aminocaproic acid (ACA) [58]. The degradation products consist of several X species, Y, D and D-dimer, as well as fragment E [59], which do not produce toxic reactions as reported in several studies [55, 60, 61]. Indeed, fibrin degradation products have been shown to possess certain biological activities, such as promoting cell proliferation, regulating cell adhesion and promoting collagen synthesis [60–62]. As for its biocompatibility, firstly, it was made of fibrinogen and thrombin from blood, and the formation of fibrin gel mimics the blood clotting process, which makes it very safe. Secondly, fibrin gel has been approved by the FDA as a haemostatic agent and adhesive for the clinical treatment of haemostasis and promoting wound healing. Thirdly, the porosity and stiffness of the formed fibrin gel can be controlled by adjusting the concentrations of fibrinogen or thrombin to meet the precise need for SCI repair. In addition, fibrin gel can also be injected as a liquid and solidifies in situ, so it can fill the lesion site in any shape. Furthermore, as shown in Additional file 1: Fig. S1, no obvious toxicity was found in fibrin gel-treated mice. Overall, the biocompatible fibrin gel with a porous structure would be a wonderful scaffold to load and release exosomes slowly in the lesion site, and all the advantages mentioned above make fibrin gel an excellent scaffold to transplant exosomes for SCI repair.

Both Gel-Exo and VGF-lentivirus exert great potential in SCI repair. According to the results of BMS in Figs. 2D and 6D, exosomes are more effective than VGF-lentivirus, mainly because of their comprehensive functions in vivo. Compared to lentivirus, exosomes are safer and more promising in clinical application, owing to their safety. In addition, exosomes could be applied as drug carriers to obtain better function. Although exosomes have shown great potential in treating SCI, lots of problems still need to be solved before exosome therapy for SCI could be used clinically. Firstly, the source of the exosomes must be determined. In addition, separation methods must be standardized and more efficient. Furthermore, the storage, preservation, and transportation of exosomes also need to be solved. Finally, further research is needed to probe the relationship between injection frequency, dosage, and the therapeutic effect of exosomes to maintain the long-term effect, which is very important for the correct use of exosomes to treat SCI. As for lentivirus, serval issues need to be emphasized. Firstly, since the vector capacity is limited, the virus titer will be greatly reduced and the demand of all target genes could not be met, when the viral vector exceeds the size limit. Furthermore, the preparation process is complicated, because the virus must be produced in the packaging cells and the virus titer must be measured. Finally, the administration methods in human body need to be discussed, since different methods can generate different therapeutic effects.

## Conclusions

**Fig. 7:**
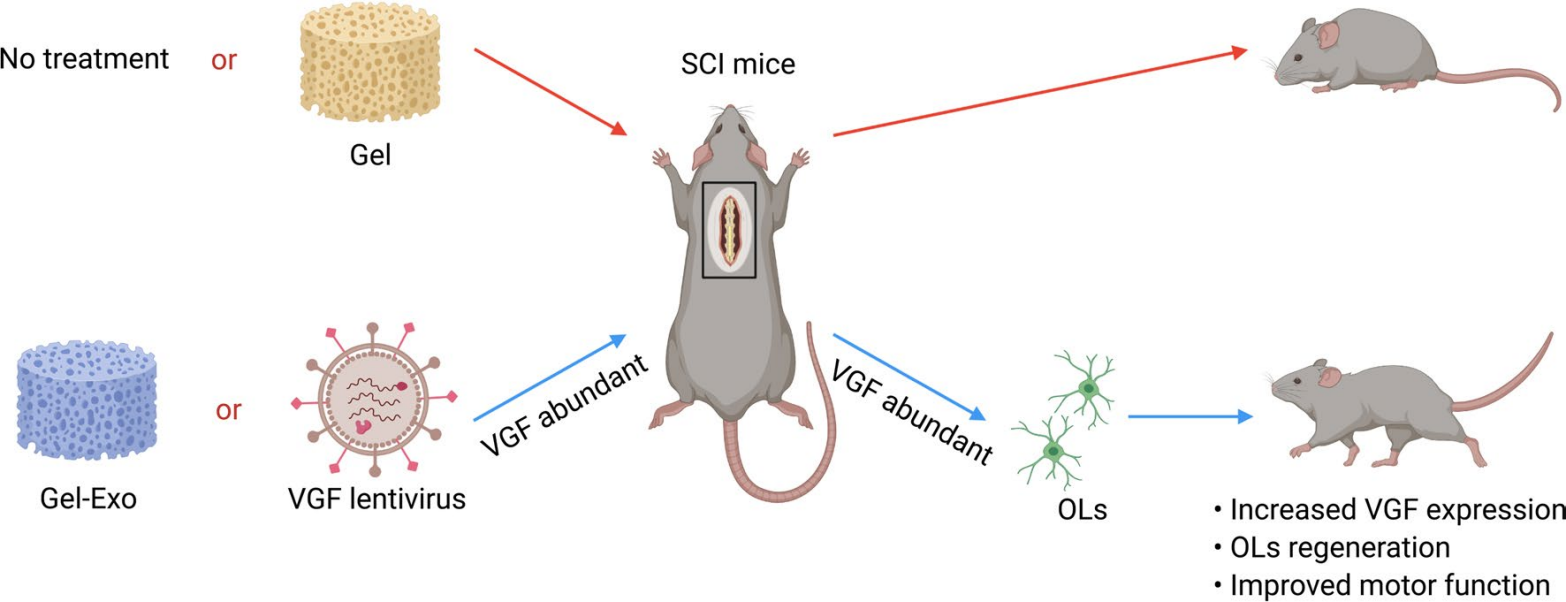
Schematic illustration of the functional mechanism of Gel-Exo in facilitating recovery after complete SCI. When Gel-Exo was introduced into SCI treatment, mice with SCI showed OPC differentiation and extensive de novo myelination, which could also be simulated by lentivirus-induced VGF overexpression in the absence of Gel-Exo. Following Gel-Exo transplantation or VGF overexpression, SCI mice displayed increased VGF expression in the spinal cord, extensive regeneration of OLs, and improved motor function. The figure was created with BioRender.com

The present research revealed a novel function of Gel-Exo in promoting behavioural performance, electrophysiological performance, and neurogenesis in mice with SCI. To further clarify the molecular mechanisms involved, we conducted transcriptome sequencing to screen for potential DEGs and found that Gel-Exo treatment significantly upregulated VGF expression. Moreover, we identified abundant VGF in exosomes, and Gel-Exo treatment with a high level of VGF promoted oligodendrogenesis. Furthermore, we employed VGF lentivirus to overexpress VGF in vitro and in vivo, which was beneficial for the differentiation of OPCs in vitro as well as oligodendrogenesis in vivo. Altogether, our results suggested that Gel-Exo or VGF lentivirus-treated SCI mice with increased VGF expression in the lesion site exhibited enhanced oligodendrogenesis and improved motor function, in contrast to the Gel-treated or untreated mice with SCI (Fig. 7). Our research provided a novel biocompatible Gel-Exo material for SCI repair, which has potential for clinical applications.

## Methods

### Cell culture

Sprague Dawley (SD) rat BMSCs and HEK 293T cells were kindly provided by Stem Cell Bank, Chinese Academy of Sciences. BMSCs were maintained in α-MEM (Gibco) containing 10% FBS (BI) and 1 × penicillin–streptomycin solution (P/S, KeyGEN), and 293T cells were grown in DMEM (Gibco) with 10% FBS and 1 × P/S.

OPCs were isolated from SD rat pups at postnatal Day 1 (P1) based on the protocol reported by Saavedra et al. [63], with minor alterations. Briefly, rats were decapitated, and the brains were quickly excised. The brain cortex tissue was then dissected, chopped into small pieces, and digested in a 37 °C water bath with papain solution. After digestion, the cell suspension was neutralized with fresh medium, filtered using a 100 μm cell strainer, and centrifuged at 1000 rpm for 5 min. The precipitate was resuspended in DMEM/F12 (Gibco) containing 10% FBS and 1 × P/S and plated into cell culture flasks. After 4–7 days of culture, the cells were digested and then purified through a differential adhesion method to remove contaminating microglia. The nonadherent cells were collected and dispensed into 12-well plates at a density of 1 × 10^5^ cells/well for further experiments.

### Flow cytometry

BMSCs were harvested using 0.25% trypsin (EDTA-free, KeyGEN) to prepare single-cell suspensions and subsequently resuspended in flow cytometry staining buffer (eBioscience). Fluorochrome-conjugated antibodies against CD11B/C, CD44, CD29, and CD45 (Additional file 1: Table S1) were added to the cell suspensions and incubated for 15 min at room temperature. After incubation, cells were rinsed twice and subjected to analysis by flow cytometry (FACSVerse, BD Bioscience).

### Exosome isolation and characterization

BMSC-derived exosomes were isolated utilizing an ultracentrifugation method. Briefly, cells seeded in 100 mm dishes were maintained in standard medium until cell confluence reached approximately 80%. Cells were then exposed to 24 h of serum starvation prior to extraction of exosomes. Next, the culture medium was collected and subjected to serial centrifugations at 300×*g* for 10 min, 2000×*g* for 10 min, and 10,000×*g* for 30 min at 4 ℃ to eliminate cells and cellular debris. Exosome pellets were obtained by subsequent centrifuging at 100,000×*g* for 90 min at 4 °C in a Beckman Optima XPN-100 ultracentrifuge. The supernatant was discarded, and the pellets were rinsed by suspension in PBS and centrifuged again at 100,000×*g* for 90 min. The resultant exosome pellets were resuspended in PBS and stored in aliquots at − 80 °C.

Exosome concentration was estimated by total protein level quantified using a BCA Protein Assay Kit (KeyGEN). The morphological characterization of exosomes on the copper net (Zhongjingkeyi) was examined via transmission electron microscopy (TEM, JEM-1230, JEOL). The particle size distributions and zeta potential were assessed with NTA (ZetaView, Particle Metrix) by VivaCell Biosciences Co., Ltd.

### Western blot

Samples were lysed on ice in lysis buffer (KeyGEN) that contained phosphatase inhibitor, protease inhibitor, and PMSF. The BCA assay was employed to measure the protein concentration following the manufacturer’s specifications. Lysed samples were then mixed with loading buffer (Beyotime Biotechnology) and boiled for 5 min. After that, 20 μg of protein for each sample was separated by SDS-PAGE (6% stacking gel and 10% separating gel) and subsequently transferred to a PVDF membrane (Millipore). Blocking was performed with 5% BSA (Sigma-Aldrich) in TBST for 1 h at room temperature. Primary antibodies diluted with 1% BSA were added to the membrane and incubated overnight at 4 °C. After extensive washing with TBST, the membrane was treated with appropriate HRP-tagged secondary antibodies for 1 h at room temperature. The antibody information is described in Additional file 1: Table S1. Protein bands were developed using ECL substrate (Millipore) and imaged with an Amersham Imager 600 imaging system (GE Healthcare).

### Fibrin gel formation and characterization

Fibrin gels (Gel) were fabricated by mixing equal volumes of 20 mg/mL fibrinogen (Sigma-Aldrich) and 20 U/mL thrombin (Sigma-Aldrich) in sterile PBS and in the presence of 50 μg/mL aprotinin (Sigma-Aldrich) and 5 mM CaCl_2_, followed by subsequent crosslinking at 37 ℃ for 10 min. For exosome-containing gels (Gel-Exo), 4 μg/mL BMSC-derived exosomes were added to the fibrinogen solution.

SEM (Gemini 300, Carl Zeiss) was applied to examine the microstructure of fibrin gels. The stress–strain curve was acquired on a universal testing machine (Z250, Zwick), and the linear region of the curve was used to calculate the compressive modulus.

### Establishment of the mouse SCI model

Female C57BL/6 mice aged 6–8 weeks, weighing 18 − 22 g, were obtained from Shanghai SLAC Laboratory Animal Co., Ltd. A complete transection model was employed in this study, and all experimental procedures were approved by the Institutional Research Ethics Committee of Tongji Hospital of Tongji University. Briefly, mice were anaesthetized via inhalation of isoflurane (1–2% in oxygen, RWD Life Science), and the surgical site was shaved and disinfected. A laminectomy was then conducted at spinal segment T8-T9 under a microscope. After the dura was opened, the spinal cord tissue was removed by gentle aspiration with a glass micropipette to form a 2 mm long lesion cavity. The mice were randomized into control and different treatment groups (n = 20 per group). In the Gel and Gel-Exo groups, fibrin gels were allowed to polymerize before transplantation to the lesion site. In the sham-treated mice, laminectomy was performed without SCI. Following surgery, bladder massage was given twice a day to help urination, and food and water were supplied ad libitum.

The body weight was recorded once a week. The recovery of motor function was rated weekly by two independent observers who were unaware of the group identity on a scale of 0–9 according to the BMS scoring criteria. For analysis of neural conduction, an electrophysiological assay was carried out 8 weeks after SCI by stimulating the brain and recording hind limb MEPs in each mouse, as described previously. Subsequently, mice were sacrificed, and the spinal cord, heart, liver, spleen, lung, and kidney tissue were collected for further assessments.

### qPCR

Total RNA was extracted from a 4 mm long spinal cord segment encompassing the lesion site using RNAiso Plus reagent (TaKaRa). After reverse transcription to cDNA with PrimeScript RT Reagent Kit (TaKaRa), qPCR was conducted on an ABI QuantStudio 3 PCR system (Thermo Fisher Scientific) with TB Green Premix Ex Taq (TaKaRa). Gene expression levels were calculated according to the 2^−△△Ct^ method and normalized to *Gapdh*. The primer sequences are listed in Additional file 1: Table S2.

### RNA sequencing

Total RNA was prepared accordingly [64], and samples were then sent to BGI Tech for library construction and sequencing on the BGISEQ-500 platform. The data obtained were analysed using BGI Dr. Tom online system. The criteria of |log2 (fold change)|≥ 1 and false discovery rate (FDR) ≤ 0.001 were used to identify DEGs. Kyoto Encyclopedia of Genes and Genomes (KEGG) pathway and Gene Ontology (GO) enrichment analyses and GSEA were performed to annotate gene function and screen key pathways.

### Immunofluorescence staining

Spinal cord tissue was fixed in 4% paraformaldehyde (PFA, Solarbio) and dehydrated successively in 15% and 30% sucrose. After being immersed in OCT embedding medium (Sakura) and frozen in dry ice, the samples were cut into 10 μm thick cryosections using a Leica CM1950 cryostat. For fixation of cultured cells, the culture medium was aspirated, and 4% PFA was added and incubated for 15 min at room temperature.

Samples were then permeabilized with 1% Triton X-100 for 10 min, blocked with 5% normal goat serum for 60 min, and labelled with primary antibodies overnight at 4 ℃. Fluorochrome-conjugated secondary antibodies were added the following day and incubated for 1 h at room temperature. After a series of washes in PBS, the samples were stained with DAPI solution to mark the nuclei. Images were collected with a Zeiss LSM880 confocal microscope.

### Plasmid construction and lentivirus administration

The full-length coding sequence of the mouse *Vgf* gene was obtained by PCR amplification using mouse brain cDNA as the template and Platinum SuperFi II DNA polymerase (Thermo Fisher Scientific) and gene-specific primers. The PCR product was then cloned into the CSII-EF lentiviral expression vector. For lentivirus production, the CSII-EF-Vgf plasmid and lentiviral packaging vectors psPAX and pMD2.G were cotransfected into 293T cells with the use of Lipofectamine 3000 reagent (Thermo Fisher Scientific). At 24 h and 52 h post-transfection, the culture medium containing lentiviral particles was collected, centrifuged at 3000×*g* for 10 min, and filtered through 0.45 μm filters to remove cellular fragments. Virus precipitation was performed by adding 5 × Lentivirus Precipitation Solution (TransGen Biotech) and incubating for 45 min at 4 °C with agitation. After centrifugation at 7000×*g* for 45 min, the virus pellets were resuspended in DMEM/F12 and kept in aliquots at − 80 °C.

A total of 1 × 10^6^ TU virus was added to fibrinogen solution, which was subsequently mixed with thrombin solution to form fibrin gels as mentioned above.

### H&E staining

For histological examination, paraffin-embedded tissue samples were sliced into 5 μm sections and stained with haematoxylin and eosin solution (Sevicebio) following standard procedures. Images were taken with an Olympus BX53 microscope.

### Statistical analysis and figure preparation

All results are presented as the mean ± standard deviation, and the statistical significance for each experiment was determined using one-way analysis of variance (ANOVA) with GraphPad Prism software. *, **, ***, and **** represent p < 0.05, p < 0.01, p < 0.001, and p < 0.0001, respectively. Raw images were assembled in Adobe Photoshop software. Scheme 1 and Fig. 7 was created with BioRender (https://biorender.com).

## Supplementary Information

**Additional file 1: Figure S1.** HE staining of the heart, liver, spleen, lung, and kidney in all groups. Scale bar, 100 μm. **Table S1.** Antibody information. **Table S2.** Primers for qPCR detection.

**Additional file 2: Video S1. Over ground walking of mice in the SCI group, Gel group, and Gel-Exo group, related to Fig. 2D. **

**Additional file 3: Video S2. Over ground walking of mice in the SCI group and VGF lentivirus group, related to Fig. 6D.**

## Data Availability

All data generated or analyzed during this study are included in this published article.
