# Supplementary material for: Biocompatible exosome-modified fibrin gel accelerates the recovery of spinal cord injury by VGF-mediated oligodendrogenesis
Source: J Nanobiotechnology. 2022 Aug 2;20:360. doi: 10.1186/s12951-022-01541-3 (PMC9344707; doi:10.1186/s12951-022-01541-3)
Supplement: Supplementary file 1 — Additional file 1: Figure S1. HE staining of the heart, liver, spleen, lung, and kidney in all groups. Scale bar, 100 μm. Table S1. Antibody information. Table S2. Primers for qPCR detection. [file 12951_2022_1541_MOESM1_ESM.docx]

**Supporting Information**

**Biocompatible exosome-modified fibrin gel accelerates the recovery of spinal cord injury by VGF-mediated oligodendrogenesis**

Xiaolie He^†^, Li Yang^†^, Kun Dong, Feng Zhang, Yuchen Liu, Bei Ma, Youwei Chen, Jian Hai, Rongrong Zhu*, and Liming Cheng*

*Key Laboratory of Spine and Spinal Cord Injury Repair and Regeneration of Ministry of Education, Orthopaedics Department of Tongji Hospital, School of Medicine, School of Life Sciences and Technology, Tongji University, 200065, Shanghai, P. R. China*

**Correspondence:* *rrzhu@tongji.edu.cn, limingcheng@tongji.edu.cn.*

^†^*These authors contributed equally to this work*


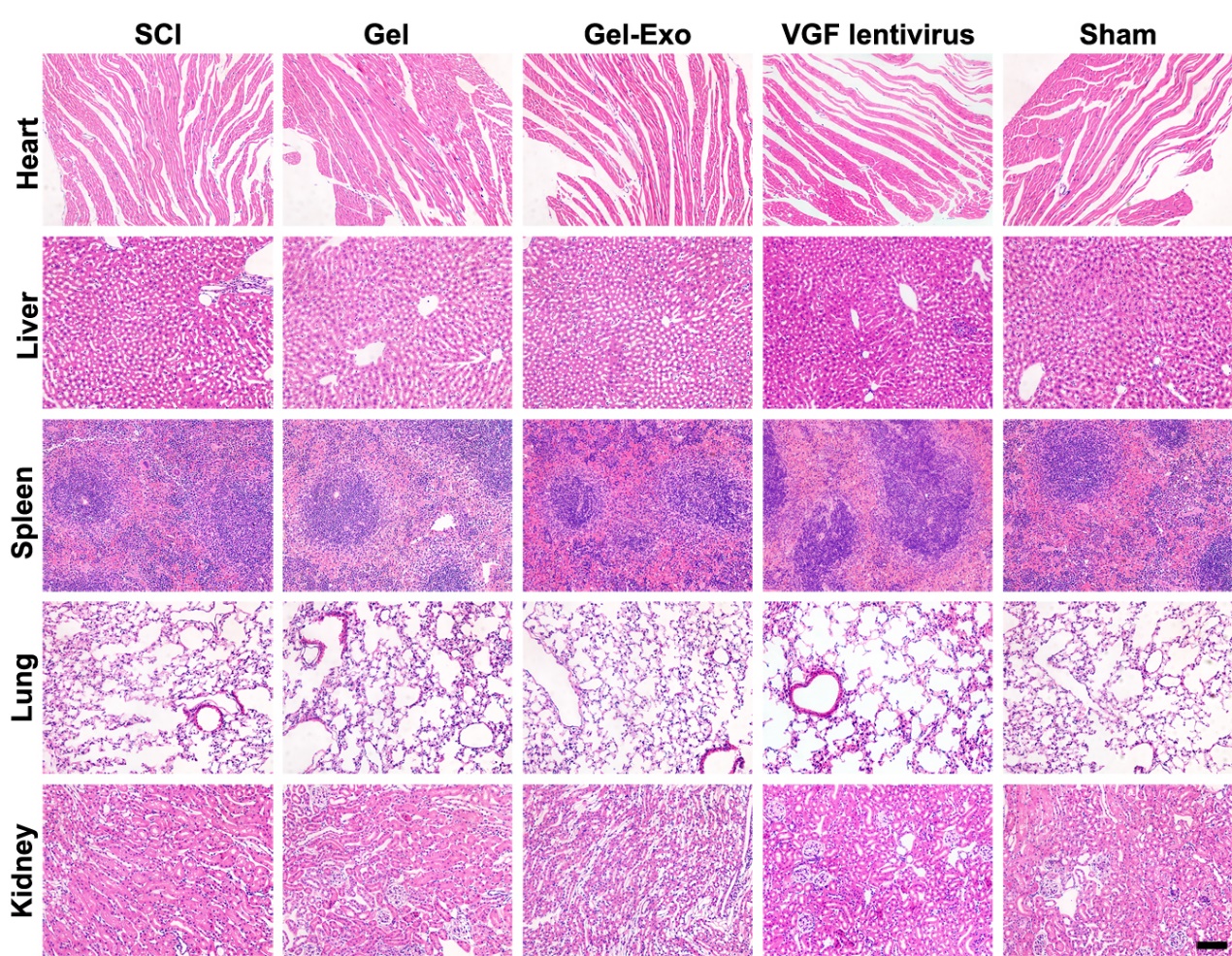


**Fig. S1** HE staining of the heart, liver, spleen, lung, and kidney in all groups.

**Table S1** Antibody information

| **Antibody name** | **Company** | **Catalog #** |
| --- | --- | --- |
| CD11B/C PE | Thermo Fisher Scientific | 12-0110-80 |
| CD44 PE | Thermo Fisher Scientific | 12-0444-80 |
| CD29 FITC | Thermo Fisher Scientific | 11-0291-80 |
| CD45 FITC | Thermo Fisher Scientific | 11-0461-80 |
| CD63 | System Biosciences | EXOAB-CD63A-1 |
| CD9 | System Biosciences | EXOAB-CD9A-1 |
| β-ACTIN | Proteintech | 66009-1- |
| NEUN | Sigma-Aldrich | ABN90 |
| TUJ1 | Abcam | ab78078 |
| VGF | Bioss | bs-11795R |
| MBP | Millipore | MAB386 |
| OLIG2 | Abcam | ab1091186 |

**Table S2** the specific surface area and pore volume

| **Species** | **Gene** | **Forward Primer** | **Reverse Primer** |
| --- | --- | --- | --- |
| Mouse | *Gapdh* | AGGTCGGTGTGAACGGATTTG | TGTAGACCATGTAGTTGAGGTCA |
|  | *Dcx* | CATTTTGACGAACGAGACAAAGC | TGGAAGTCCATTCATCCGTGA |
|  | *NeuN* | GGGTATGGGTAGGATTGGGG | GTGGAAGGTTTCACTACAACAGA |
|  | *Tuj1* | TAGACCCCAGCGGCAACTAT | GTTCCAGGTTCCAAGTCCACC |
|  | *Vgf* | AAGGATGACGGCGTACCAGA | TGCCTGCAACAGTACCGAG |
|  | *Mbp* | GGCGGTGACAGACTCCAAG | GAAGCTCGTCGGACTCTGAG |
|  | *Olig2* | TCCCCAGAACCCGATGATCTT | CGTGGACGAGGACACAGTC |
|  | *Sox10* | ACACCTTGGGACACGGTTTTC | TAGGTCTTGTTCCTCGGCCAT |
| Rat | *Gapdh* | AGTGCCAGCCTCGTCTCATA | TGAACTTGCCGTGGGTAGAG |
|  | *Vgf* | CAGCCCTCGACCATCTTTCA | CGCAATCCTCGTTCTGGGTA |
